# Gastronomy Tourism and Well-Being: Evidence from Taiwan and Catalonia Michelin-Starred Restaurants

**DOI:** 10.3390/ijerph19052778

**Published:** 2022-02-27

**Authors:** Min-Pei Lin, Estela Marine-Roig, Nayra Llonch-Molina

**Affiliations:** 1 Department of Specific Didactics, University of Lleida, 25003 Lleida, Spain; lm16@alumnes.udl.cat (M.-P.L.); nayra.llonch@udl.cat (N.L.-M.); 2 Department of Business Administration, University of Lleida, 25003 Lleida, Spain

**Keywords:** gastronomy tourism, gastronomic image, customer expectations, subjective well-being, service quality model, user-generated content, online travel review, TripAdvisor, Taiwan, Catalonia

## Abstract

In the tourism and hospitality industry, ensuring the well-being of visitors is essential to achieving a competitive tourist destination. This objective is even more pressing in the gastronomy sector. Surprisingly, the scientific literature on this topic is scarce and relies on questionnaire surveys and interviews as a data source. After scrutinizing the 13 articles on gastronomy tourism and well-being indexed in the Web of Science or in Scopus, this study proposes two new lines of research interrelated by the concept of gastronomic image. These exploit the content shared online by consumers in order to assess subjective well-being derived from quality gastronomic experiences. The first is a framework for the customer-perceived image based on Grönroos’s service quality model, and the second is a conceptual model based on Morris’s semiotics to measure gastronomic image. Through mixed methodologies, i.e., qualitative in the first research line and quantitative in the second, the study applies the theoretical framework to Michelin-starred restaurants in two tourist regions with similar features but with different gastronomic cultures—Taiwan (Asia) and Catalonia (Europe)—using as a data source all the online travel reviews (OTRs) written in English about these restaurants shared on the TripAdvisor portal. Comparing the three categories of restaurants in both regions, the results show branding and marketing problems and significant differences in the popularity of restaurants and the satisfaction and well-being of diners. There is a positive relationship between the category of restaurants according to the number of Michelin stars and their popularity according to the number of OTRs, as well as with the satisfaction and well-being of diners, except for a 3-star restaurant that is the worst-rated. These outcomes from the demand side can be useful to stakeholders to design or improve gastronomic products and services.

## 1. Introduction

Gastronomy tourism is a type of activity characterized by the visitor’s experience linked with food-related products and activities, such as authentic, traditional, and/or innovative culinary experiences [1]. Increasingly, more destinations are emphasizing that type of cultural tourism [2] because food largely impacts not only the health of tourists, but also their overall well-being [3]. Faced with this growing interest in gastronomic tourism, many famous chefs from high-end restaurants focus their creative cuisine on the combination of cuisine, territory, landscape, and culture [4], thus recognizing the value of the local gastronomic heritage [5].

For many scholars, well-being seems to be synonymous with quality of life [6]—or they are at least two closely related concepts [7]. Felce [8] understands quality of life as a person’s overall well-being, which is determined by the combination of three factors: objective life conditions, subjective well-being (i.e., personal satisfaction with life conditions), and personal values and aspirations (i.e., the importance given both to objective life conditions and personal satisfactions). The author also establishes a correlation between personal satisfactions and subjective well-being. Although well-being is intangible, difficult to define, and even harder to measure, it is a goal pursued by various countries, regions, and even individuals. In addition, well-being is strongly subjective; each person seeks it differently; that is, in the face of the same situation, people have different levels of well-being.

People face all kinds of pressures in a modern society characterized by a rapid pace of life. Added to this is the economic recession caused by the COVID-19 pandemic and the resulting demands for social distancing, which have altered the daily lives of the global population [9]. Such a large and sudden disturbance to daily life is likely to have a major negative impact on human well-being, especially for those living in densely populated cities. A recent study [10] of 823 full-time workers in Taiwan revealed that a simple hotel stay provides a respite from work and reduces work-related rumination, thereby contributing to guest satisfaction and both hedonic and eudaimonic well-being. These invisible life pressures make people want to capture a little happiness in their lives, and eating delicious food in a restaurant is one expedient way of meeting this need. For consumers, revisiting the happiness of a gastronomic experience is usually one of the main factors that makes them repeat customers [11].

Nowadays, because of the increasing socialization and diversification expectations of customers, the restaurant has become a place where customers can receive not only food but also a complete gastronomic experience [12]. Fine dining is a highly creative and innovative industry, always full of challenges and self-requirements, providing guests with a unique gastronomic experience [13]. A Michelin-starred restaurant can be synonymous with fine dining. Indeed, the reputation of fine dining or haute cuisine has increased, in part, due to the appearance and expansion of the *Michelin Guide*, which makes fine-dining cuisine more international and diverse and encourages the pursuit and enthusiasm of unique and unforgettable gastronomic experiences that provide tangible and intangible products and services [14]. The Michelin company published the first *Michelin Guide* in France in 1900, and it has been updated every year since. Restaurants around the world aspire to achieve the professional and rigorous standards of Michelin-starred restaurants in order to gain recognition and enhance their competitiveness [15]. Michelin-starred restaurants are an important base from which people can pursue quality gastronomic experiences.

Traditionally, most researchers obtain data through questionnaire surveys and interviews in order to obtain information on the satisfaction of restaurant customers [5,16]. These methods can not only highlight important themes and enable the testing of specific hypotheses but also provide richer insight data. New information and communication technologies (ICT) currently make it possible to obtain massive amounts of data on customer or consumer satisfaction through user-generated content (UGC). Both data sources, survey and UGC, are complementary [17]. An increasing number of researchers use UGC as a data source, which gives much more information with less investment of resources and is based on the already investigated premise of the potential of word-of-mouth (WoM) marketing—meaning that most consumers believe more in UGC than in producer-generated content—for both positive and negative information [18]. Due to the great progress of ICT and the popularity of travel review platforms with diversity and user-friendly presentation, sources of WoM or digital WoM (eWoM) on review platforms have gradually become popular for tourists and academic researchers, and the number of users has increased exponentially. One of these travel-related platforms based on UGC is TripAdvisor, which was founded in the early 21st century and became the world’s most popular travel destination and accommodation website in a short period of time. TripAdvisor currently hosts more than 988 million reviews and comments shared online by travelers. Such a huge amount of information has naturally become a source of research data for gastronomy-related studies [9,19,20,21].

Studies linking gastronomic tourism and well-being are scarce and, among them, the use of UGC as a source of research data is rare. To fill the research gap, this study proposes a new theoretical and methodological framework to understand the gastronomic image perceived by customers and measure their satisfaction, loyalty, and well-being through content generated by consumers and shared on social media. The chief purpose of the research is to confirm, using tourist UGC as data, that consuming quality food contributes to diners’ satisfaction and subjective well-being. The theoretical framework includes two interrelated conceptual models to support a mixed analysis methodology. The qualitative method is based on the Grönroos [22] service quality model through technical quality, functional quality, and service environment, integrated into the hermeneutical circle of image formation [23], which results in an image perceived as the quality of the product, service, and atmospherics [24]. The quantitative method is based on a semiotic model that adapts Morris [25] trichotomies to the study of gastronomic images. This theoretical approach aims to contribute to future research on gastronomy tourism and well-being because the new integrated framework has a solid foundation and enables the exploitation of UGC as a data source. The study applies the theoretical and methodological frameworks to a case study to test the proposal [26]: Michelin-starred restaurants in two regions with similar settings, but with different food cultures—Taiwan in Asia and Catalonia in Europe—using all online travel reviews written in English and shared on the TripAdvisor portal as a data source. The outcomes of comparing customer opinions on the three categories of restaurants in both regions can be useful to stakeholders in designing or improving their products and services from a demand-side perspective.

## 2. Theoretical Background

Although gastronomy can bring happiness and become one of the purposes of travelling, gastronomy cannot provide a complete or lasting food experience [27]. What is needed to complete a pleasant gastronomic experience comes from the perfect match between the food and the surrounding material and immaterial aspects; food is only a part of those elements. In order to develop the various aspects of gastronomic experiences and well-being, this section is divided into five parts: gastronomy and well-being relationships; a review of the literature relating to gastronomic tourism and well-being; consumer satisfaction and well-being through quality experiences; perceived gastronomic image; and fine dining restaurants.

### 2.1. Relationships between Gastronomy and Well-Being

Perceived service quality is an antecedent of customers’ subjective well-being [28]. In other words, a high level of positive evaluation of the specific quality of goods and services represents a higher level of consumer well-being [29]. Although the epicurean consumption of food contributes to improving the consumer’s well-being [30], the literature on gastronomy and well-being remains limited. Even so, through surveys, several researchers have demonstrated various relationships between food-consumption experiences and the subjective well-being of consumers, as shown by the following conclusions: the culinary experience affects psychological well-being (524 usable surveys) [11]; the effects of foods on well-being are strongly related to physical health, pleasure, and emotional aspects (755 participants from five countries) [31]; on-site meals, dinner and breakfast, contribute to holiday well-being (243 respondents) [32]; there is a significant positive correlation between food-consumption motivations and the well-being of “foodies” (480 valid responses) [33]; and finally, tourists with food neophobia experience positive effects on their well-being when consuming comfort food (381 valid formal surveys) [34].

### 2.2. Gastronomy Tourism and Well-Being Literature Review

In the last few decades, researchers in the field of tourism have shown a growing interest in the study of psychological well-being, focused mainly on the subjective well-being of tourists [6,35,36], to the point of considering well-being as a tourist resource [37]. In order to analyze the research published so far on gastronomy tourism and well-being, a systematic literature review was carried out using the PRISMA guidelines [38]: 25 records were identified (12 Web of Science and 13 Scopus) through bibliographic database searching (Box 1); 14 records remained after duplicates were removed; 13 studies were included in review (one study was excluded as it was not related to tourism).

Box 1 **Boolean search formulas for gastronomy tourism and well-being terminology.**

Scopus:

DOCTYPE(ar OR re) AND TITLE((well-being OR wellbeing) AND (gastronom* OR food* OR wine* OR restaurant* OR cuisine OR culinary OR breakfast OR lunch* OR dinner OR dining OR dine*)) AND TITLE-ABS-KEY(touris* OR hospitality OR destination*)

Web of Science (WoS Core Collection SCI-E, SSCI, and A&HCI):

DT = (article OR review) AND TI = ((well-being OR wellbeing) AND (gastronom* OR food* OR wine* OR restaurant* OR cuisine OR culinary OR breakfast OR lunch* OR dinner OR dining OR dine*)) AND TS = (touris* OR hospitality OR destination*)

Total of 13 articles are described in Appendix A (Table A1) and indexed in the Web of Science (WoS) or Scopus bibliographic databases, which study the relationships between gastronomy and well-being in the field of tourism and hospitality, showing that data are scarce and based on the analysis of questionnaires and interviews, except for one that scrutinizes available literature. Almost all of the articles are recent (as of 2020), showing that the topic was previously not of significant interest to researchers. Instead, this study proposes a new line of research, through new theoretical and methodological frameworks, that exploit the content shared online by customers or consumers.

### 2.3. Consumer Satisfaction and Well-Being through Quality Experiences

**Figure 1:**
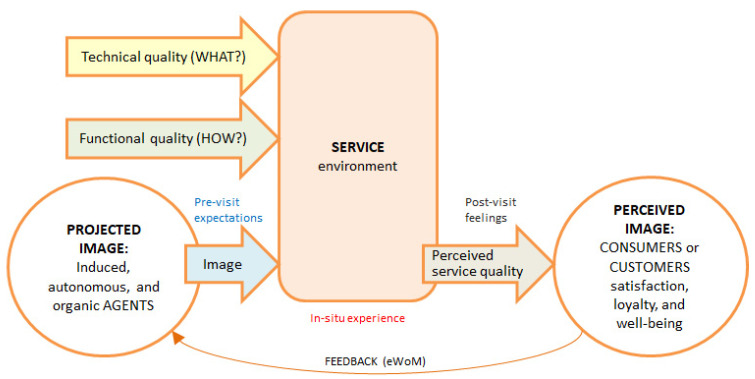
Subjective well-being formation underpinned by previous works [22,39].

In the field of hospitality and tourism, numerous authors have identified the relationship between the destination image and visitors’ satisfaction and loyalty [23]. However, whether any experience in particular contributes to the well-being of tourists has not been shown. Instead, several authors demonstrated these relationships in the context of the consumption of quality goods and services [28,29]. For that reason, the new conceptual model (Figure 1) is based on Marine-Roig’s hermeneutic circle of destination image formation [23] and Grönroos’s service quality model [22]. In short, the proposed model addresses quality experiences in the context of hospitality and tourism, and this study uses it to analyze consumers’ subjective well-being resulting from dining experiences in luxury restaurants.

#### 2.3.1. Circle of Destination Image Formation

Marine-Roig considers the destination image as a gestalt, a whole that differs from the parts that comprise it and represents image formation through a hermeneutic circle [23,39]. At one extreme are the agents who project the image. Simplifying Gartner’s model [40], the agents or sources of information can be induced, autonomous and organic [41]. Induced sources depend on destination promoters (e.g., destination marketing or management organizations, and tour operators) including tourism service firms or their brands (e.g., Ritz, Starbucks). Organic sources come from individuals and are spread through word-of-mouth marketing (WoM) or UGC (eWoM). Finally, autonomous sources are independent of the previous two (e.g., travel writings, and guidebooks). At the core of the framework is the visitor experience. At the other end of the diagram is the image perceived by visitors, which is conditioned by the expectations generated by the projected image.

#### 2.3.2. Service Quality Model

Grönroos [22] indicates that customer experience contains three crucial elements: technical quality, functional quality, and image as a quality (Figure 1). Technical quality refers to material aspects, while functional quality and image are related to immaterial aspects. For customers, immaterial experiences have become an important factor to consider, for example, when eating in a restaurant because tasty meal is already a basic requirement for a quality dining experience. In summary, the Grönroos model has three inputs –technical quality (what?), functional quality (how?), and image– and one output –perceived service quality. This outcome depends on the comparison of two variables, namely the expected service and the perceived service. That is, the image or brand of services generates consumers’ pre-visit expectations, and in the on-site experience they subjectively perceive the service received. The customer’s post-visit feelings correspond to the perceived service quality.

Technical quality has to do with the material aspects the customer perceive, mainly in the form of the product that he or she acquires or consumes, and is the technical result of the process that has created it [22]. In the case of a restaurant’s service, the outcome of the process that the customer receives is a meal. This cuisine product is the material aspect that most directly link to technical quality, while there are also other intangible aspects, such as taste, aroma, and appearance, which directly affect the emotion of customers [42].

Together with technical quality, which has to do with “what” the customer gets, customers also perceive what is called functional quality, which has to do with “how” they get the technical outcome; that is, “functional quality corresponds to the expressive performance of a service” (p. 39) [22].

In the case of gastronomic experiences, in order to make it perfect, the service generated in response to the food experience must be in keeping with the following three conditions: the customers’ perception being satisfied (aesthetic experience); the meanings we attach to the products (experience of meanings); and the feelings and emotions evoked by customers (emotional experience) [43]. To accomplish these goals, the servicescapes of the service industry are divided into three dimensions: environmental conditions; space/function; and signs, symbols, and artefacts [44]. Other authors [45] proposed dinescape, a six-factor scale that separates the physical environment and service of the dining environment to measure facility aesthetics, ambience, lighting, service product, layout, and social factors. However, these two categories—servicescapes and dinescape—are not complete for all service industries [46]; in addition to food, service, and atmosphere, it is also considered a “user imagery” formed by other restaurant customers [47].

According to the stimulus-organism-response (SOR) theory [48], after customers enter a restaurant they develop corresponding reactions and behaviors based on their perception of the restaurant environment to express their internal emotions. The source of emotions includes all the contents of servicescapes [44], which means the sum of customer experience is related to the customer’s expectations of the restaurant theme and the quality of the presentation. Servicescapes include a variety of details that affect customers’ experience. For example, pleasant music can help increase customer’s consumption time compared to less pleasing music [49].

#### 2.3.3. Restaurants Image as a Quality

As lifestyle changes, dining out becomes more and more common, and customers desire new flavors, comfortable atmospheres, and pleasant memories. More importantly, they prefer an excellent overall dining experience, which is composed of tangible and intangible elements [50]. Previous studies have found that the perceived quality of the physical environment [51] or the quality of service [52] can significantly affect the business image. Those business images are gradually accumulated by consumers based on their personal consumption experiences and word of mouth and thus form part of brand equity [53]. Brand equity endows value on products via branding, can be perceived from the perspective of the industry, trade or consumer before being introduced to customers, and is often used to protect the business [54]. By contrast, a company’s brand equity is the basis for customers’ consumption-related expectations. Therefore, the restaurant industry presents restaurants based on an image of theme aesthetics, which, together with the brand, name, and symbol, form a brand equity that is distinguishable in the industry [55] and that becomes the basis for the design of servicescapes. This image will have a subsequent impact on customers’ perceived value and satisfaction, which in turn affects their loyalty [56]. From the viewpoint of customers, brand equity is based upon the value of the associations in the mind of the consumer, which may be recalled upon presentation of the brand, name, or symbol. Brand associations are what hold the value rather than the name and/or symbol itself.

The servicescapes provided by the restaurant industry include items that are easy to manage (such as lighting, table setting, physical environment for the layout of the meal), as well as items that are not easy to manage (such as the consistency of food quality, the service attitude of the staff, the correct delivery of meals in the right time, and the environment formed by other customers). Different customer approaches (such as prices and discounts) will shape the perception of the services provided by the industry.

#### 2.3.4. Dimensions of the Perceived Quality of Restaurant Services

Homogenization continues to increase in the global competitive tourism market, and it is not easy to reach the market segmentation target; therefore, those products or services that are able to satisfy the needs of the clients and industries will obtain important competitive advantages. In this service-oriented era, consumer participation is necessary in the process of understanding consumer needs, and only consumer participation can make their real needs understood [57]. That is to say, the product or service improvement before and after consumption is no longer a one-sided job of the service industry, but requires the joint creation of consumers.

**Figure 2:**
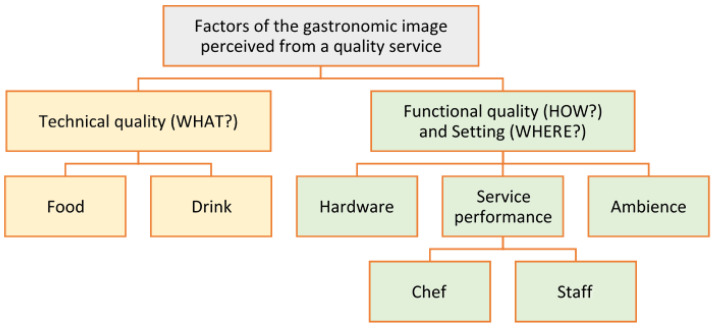
Gastronomic image perceived by diners in quality restaurants (own elaboration).

Figure 2 shows detail of the elements and sub-items of Figure 1 that make up the gastronomic experience of restaurant customers. The food (e.g., main course, side dish, and desert); drink (e.g., wine/sake/tea pairing); hardware (e.g., furniture, decoration, crockery, and cutlery); ambience (e.g., atmosphere, layout, light, and music); service performance: attitudinal aspects (e.g., smiling and friendliness of staff, professional and talented chef); social aspects (e.g., interaction with customers when staff express their knowledge of the food and culture, and when the chef shows gratitude and welcomes customers’ visit). In a nutshell, the items in Figure 2 correspond to a quality on-site dining experience, that is, the “Service environment” from Figure 1, including “Technical quality” and “Functional quality”.

### 2.4. Perceived Gastronomic Image Online

According to Chang and Mak [58], the gastronomic image from the perspective of tourists can be defined as “tourists” holistic impression about a destination’s gastronomic products and food culture” (p. 91). Despite the abundance of literature on destination image [59,60] and the growing interest of destination marketing organizations (DMOs) in promoting local gastronomic heritage [5,61], there is a paucity of research regarding the importance and implications of gastronomy tourism on destination image formation [62].

Tourism destination image (TDI) definitions [63,64] have a strong semiotic component [65]. The image is made up of subjective perceptions of those unusual or even unique attributes, attractions, or services of destinations. When a visitor shares on the social media a photo of the Longshan Temple in Taiwan or the Basilica of the Sagrada Familia in Catalonia, he or she not only spreads a religious building but a symbol of the tourist destination. The same happens with a gastronomic image. Diners post photos of typical local dishes or creative dishes from upscale restaurants contributing to form the online image of the tourist destination [66], along with other variables such as the atmosphere of the place and the quality of service that shape their satisfaction and well-being. A meal, in addition to being a consumer support experience, is a peak experience [67]. The semiotics of consumption are related to emotions and can provide hedonic and aesthetic benefits [68,69].

**Figure 3:**
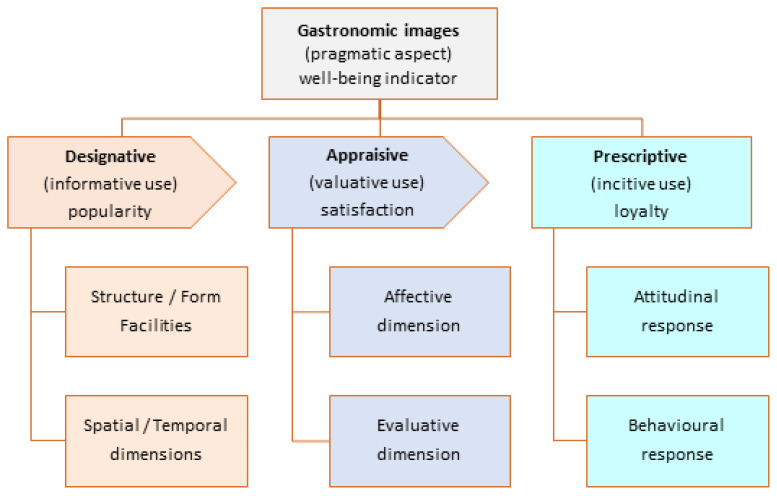
Semiotic aspects of gastronomic images derived from a previous work [23].

Thus, the deep semiotic constituent of the perceived image underpins its analysis through semiotic conceptual models fully contrasted in other scientific fields [70]. Figure 3 shows an adaptation of the philosopher and semiotician Charles William Morris’s trichotomies [25,71] to the semantic—designative, appraisive, and prescriptive—and pragmatic—informative, valuative, and incitive—aspects of the gastronomic images [21]. As Figure 3 shows, the three semantic aspects are distinctly different but hierarchically interrelated [68].

Scholars agree on the positive relationship between consumer satisfaction and well-being perception [72]. That is, when the level of satisfaction increases, the level of consumer well-being also increases. Therefore, consumer well-being can be described as consumer satisfaction from the consumption of a high-quality good or service [73], which implies optimal experience and functioning [74]. In addition, quality relationships influence customer satisfaction and loyalty formation [75]. Thus, experiences, positive feelings, and satisfaction are key elements of consumer well-being [76]. Customer satisfaction is significantly determined by customer expectations and offer performance, which become the powerful drivers of customer loyalty [77]. In summary, overall consumer satisfaction fully mediates the relationship between perceived service quality and the loyalty and subjective well-being of tourists [28]. Figure 3 also depicts the variable popularity of experiences, which quantitatively impacts the other two variables of consumer satisfaction and loyalty.

The informative use of the designative aspect answers the questions what, where, and when. In other words, it facilitates the classification of tourist experiences, locating them in time and place, and deduces their popularity from the number of comments received. In this case study, the designative semantic aspect corresponds to the “Service environment” (Figure 1), that is, the description of a quality gastronomic experience and its surroundings. The appraisive aspect has two dimensions: affective and evaluative. The affective dimension measures consumers’ feelings and moods, considering the positive and negative polarities of these sentiments. The evaluative dimension consists of scoring the experience on a scale that ranges from the worst to the best. The valuative use of the appraisive dimension determines consumers’ satisfaction. The prescriptive dimension has two dimensions that determine the attitudinal and behavioral loyalty of consumers. In summary, the model provides three metrics: the popularity of experiences and the satisfaction and loyalty of consumers, which are indicators of their well-being in the experience environment.

Regarding the model proposed in Figure 3, UGC is the ideal data source to elucidate the gastronomic image; specifically, travel blogs and online travel reviews (OTRs) shared in social media by visitors, customers, or consumers. Travel blogs and OTRs are narratives, opinions, images, and ratings freely shared on travel-related portals by visitors, based on their in situ experiences related to tourism activities, goods, and services [78]. For example, experiences in farmers markets, wine cellars, cooking courses, and dining in restaurants. This data source was used in the hospitality industry, especially to study lodging-related topics [79,80], but its use in gastronomic heritage research was rare [5]. It is worth highlighting its use in previous works to measure the gastronomic image online [20], analyze dietary needs [81], analyze diners’ sentiments [9], and compare gastronomic activities other than dining in restaurants [21]. Nonetheless, using UGC as the only data source has limitations in regards to highlighting important themes, verifying specific hypotheses, and providing insight into the richness of the data, which needs to be supported by other research methods [17].

### 2.5. Fine Dining Restaurants

Dining in luxury restaurants is not done only to solve basic physiological needs, but also to experience differentiation and hedonism [82]. Diners at these restaurants fall into the category of “experiencers”, who regard food as an essential factor in selecting a holiday destination [83]. Previous studies have shown that the consumption and services of luxury goods are a symbol of consumers’ personal identity and social status [84], which can improve personal self-assessment and define personal social class and cultural boundaries [85]. Even more, people usually show wealth through leisure activities and luxury services, which makes them equate price with product quality and even consider it as a status symbol [86].

The paradigm of fine dining restaurant is the Michelin star, and people able to spend in Michelin-starred restaurants are naturally considered to have wealth, social status, or prestige [87]. In any case, in addition to the aforementioned reasons, both regular and sporadic restaurant customers seek a complete and global experience that can be defined in terms of maximum quality with respect to the concepts seen in Figure 1 and Figure 2 when going to a Michelin-starred restaurant. If everything goes well and customers perceive the highest quality that Michelin-starred restaurants are supposed to give, the satisfaction obtained will be high and their well-being will increase. Conversely, if the experience is not entirely satisfactory, the feeling of well-being diminishes because the expectative of a Michelin-starred restaurant is high.

## 3. Materials and Methods

The case study is based on opinions shared online by customers of Michelin-starred restaurants, because the consumption of quality goods or services is a determinant of the well-being of users or consumers, as shown in Section 2. The methodology for collecting and analyzing the OTRs is an extension of previous studies [21,23]. The quantitative analysis is based on the semiotic conceptual model of Figure 3 and uses the metrics defined in a previous work [78]. The qualitative analysis consists of applying the conceptual model of Figure 2 to a random sample of OTRs segmented by content (model items) and opinion polarity (positive or negative). To perform a comprehensive qualitative data analysis (QDA) in a timely manner, software tools such as MaxQDA, ATLAS.ti or NVivo are necessary.

### 3.1. Case Study: Taiwan and Catalonia Michelin-Starred Restaurants

One located in Asia and the other in Europe, Taiwan and Catalonia are two territories of similar size with two distinctive cultural traditions that serve as attractions for visitors; therefore, they share features that facilitate their study and comparison. Whereas Taiwan has become an emerging tourism market in the Asia-Pacific region, in part owing to the country’s richness and variety of natural and cultural resources. Catalonia has long been one of the main tourist destinations in Europe owing to assets such as culture, nature, sports, and business. Tourism in both regions was one of the economic sectors most affected by the pandemic. Taiwan received 7.5 million foreign tourists in 2019 and 1.1 million in 2020 [88]. Catalonia received 19.4 million foreign tourists in 2019 and 3.9 million in 2020 [89]. As an indicator of the importance of gastronomy tourism in Catalonia, the catering sector had both an operating income and a production value of more than €10,000 million in 2019, which was double the number in the accommodation sector [90].

The *Michelin Guide* has designed food evaluation into a set of standards that can be quantified and evaluated, becoming a goal pursued by high-end restaurants all over the world. The five criteria of Michelin stars awarded to restaurants are (1) quality of products, (2) mastering of flavors and cooking, (3) personality of the cuisine, (4) value for money, and (5) consistency [91,92]. These standards are only for food; that is to say, the standard of techniques and gastronomy is the art of the chef [93]. The chefs of high-end restaurants, especially Michelin-starred restaurants, must have intuition, aesthetic sensitivity, and professional knowledge [94]. The chef plays the role of artisan and artist and must ingeniously process and combine ingredients that cannot be easily copied by others [95].

### 3.2. Data Collection

TripAdvisor, a well-known and popular travel-related website, offers both tourists and academics information on tourist destinations that can be important research data [78]. As discussed in previous sections, the UGC in the form of OTR is of special interest to researchers, since OTRs provide a quantity of information that is difficult to obtain by other means; in turn, this information is mediated by external agents but emanates directly from the user experience [78]. To carry out the research, the UGC was compiled in the form of the OTRs collected on TripAdvisor about gastronomic experiences in Taiwan and Catalonia. More specifically, all TripAdvisor OTRs in English from the “Restaurants” section related to Michelin-starred restaurants in Taiwan and Catalonia were downloaded.

According to the *Michelin Guide*, there are 34-star restaurants in Taiwan and 49 in Catalonia; three of the Taiwanese restaurants were not registered on the TripAdvisor website and for another two there were no English reviews as of the date of data compilation for this research. Therefore, the total OTRs for 29 Taiwanese restaurants and 49 Catalonian restaurants were collected. In both regions there were opinions about the three categories of Michelin-starred (1-star, 2-star, and 3-star) restaurants. Only English reviews were downloaded through a website copier with appropriate filters, so that 1038 OTRs from Taiwan and 7138 OTRs from Catalonia were obtained.

### 3.3. Data Arrangement

Through techniques developed in previous works [96,97], a text editor suitable for implementing regular expressions (search and replacement patterns) extracted all significant information from TripAdvisor OTRs and stored it in a CSV file (comma separate values). The file contained a record for each OTR with the following fields: geographic code of the resource and location of the restaurant, code of the resource and name of the restaurant, category of the restaurant, code of the OTR, score from reviewers, date and language of the OTR, and title and textual content of the OTR.

### 3.4. Content Analysis

Content analysis includes techniques for converting symbolic data to a format suitable for statistical analysis. Based on natural language processing (NLP) techniques [98], quantitative analysis uses frequency of key terms grouped by categories. The most frequent key terms arouse the most interest [99], and, according to words with close meaning and connotation, they could be categorized exclusively. To analyze the gastronomic image of a dining experience as perceived by customers, three semantic aspects (designative, appraisive, and prescriptive) of quantitative content analysis (based on categories, metrics, and rankings) were considered. The aggregations of rankings are based on the method proposed in a previous work [23]. Considering several ordered lists of N metrics, the method awards points according to the position occupied. For example (Table 1), in the case of 5 metrics with positive polarity (+), the first candidate gets 4 points, the second 3 points, and the last 0 points; if the metrics have negative polarity (−), the first candidate gets 0 points, the second 1 point, and the last 4 points; the sum of points determines the final ranking. In the event of a tie in the sum of points, the method assigns an intermediate position. For example, if the tie is between the second and third positions, the method assigns position 2.5 to both.

Considering the qualitative analysis, the categories include key terms related to the items of the model in Figure 2, considering that an OTR can include comments on different items (e.g., food, drink, chef, and staff). The pragmatic uses of the conceptual model in Figure 3 determine visitor satisfaction and loyalty, quantitatively enhanced by the popularity of the tourist resource. The set of the three variables is a determinant of consumer well-being.

#### 3.4.1. Designative Aspect

The design aspect includes the structure or form and the facilities of the tourist resource. It also considers the spatial and temporal dimensions of resources. The OTRs contain enough information to identify the resource and place it in time and space. There may be inconsistency with the time dimension due to lag between the visit and publication dates of the OTR [100,101]; that is, between the perceived and projected images. This study only considers the publication date because the OTR can be consulted by any user at any time. The number of OTRs indicates the popularity of each tourist resource.

#### 3.4.2. Appraisive Aspect

Customers leave a rating score of one to five bubbles for their gastronomic experience on TripAdvisor with a comment. In the evaluative dimension, the study applies a weighted average score (0–100) calculated for the ratings of one-to-five bubbles on TripAdvisor and categorized as follows: positive score (Score+), 5* = Excellent (100) and 4* = Very good (75); neutral score, 3* = Average (50); and negative score (Score−), 2* = Poor (25) and 1* = Terrible (0). Regarding the affective dimension, positive feelings and moods (Feel+) are considered in the textual elements of the reviews, such as “amazing” and “excellent”; and negative feelings and moods (Feel−), such as “bad” and “disappointing”, are analyzed. The pragmatic valuative aspect of this construct allows deducing user or consumer satisfaction.

#### 3.4.3. Prescriptive Aspect

The prescriptive aspect, which represents tourists’ loyalty, includes attitudinal and behavioral responses to previous stimuli. With attitudinal responses, the categories of positive attitudes “Recom+” (e.g., must not miss) and negative attitudes “Recom−” (e.g., not recommend) show whether tourists recommend or discourage the experience. For behavioral intentions, positive intentions “Behav+” (e.g., return next time) and negative intentions “Behav−” (e.g., not be back) express users’ willingness or unwillingness to (re)visit the restaurant [102].

#### 3.4.4. Factors of the Gastronomic Image Perceived from a Quality Service

Regarding the constructs of the conceptual model of Figure 1 and Figure 2, although the analysis methodology is qualitative, the categories include some key terms that facilitate a preliminary segmentation of the OTRs. The categorization is neither exclusive nor exhaustive, because an OTR can contain unexpected comments and others related to various constructs. Regarding the positive or negative polarity of the opinions, the appraisal aspect metrics seen above allow a rough classification of the OTRs.

The presentation of quality comes from the content and number of positive and negative comments, with the following examples. (1) Technical quality: positive comments (e.g., “outstanding food”, “beautiful presentation”, “fresh product”); and negative comments (e.g., “portion so small”, “food mediocre and not hot”, “wine pairing also did not match”). (2) Functional quality: positive comments (e.g., “beautiful wooden locker”, “ample space between the tables”, “pleasant ambience”); and negative comments (e.g., “bad décor”, “dirty floor”, “no tablecloths”). (3) Service performance: positive comments (e.g., “talented chef”, “knowledgeable staff”, and “attentive service”); and negative comments (e.g., “staff did not smile”, “service robotic”, and “chef did not come and greet the table”). (4) Perceived service quality: positive comments (e.g., “highly recommend”, “worth every penny”, and “definitely return”); and negative comments (e.g., “far from expectation”, “over-priced”, “will never go back”).

## 4. Results and Discussion

Table A2 in Appendix B shows the forty most frequent key terms per region sorted by percentage of the total number of words (including stop words) in restaurant OTRs. As expected, the most frequent keywords in both cases are “food” and “restaurant”. It should be noted that the keyword “wine/s” is more frequent in Catalonia, with twice as many occurrences as a percentage than Taiwan. This result confirms that wine consumption is more common in European countries than in Asian ones [21]. The capital of Taiwan, Taipei, is in position 7 (0.29675%) of the ranking, while the capital of Catalonia, Barcelona, is in position 15 (0.19838%). Regarding the visitor’s knowledge of the brand of the tourist destination and the identity of its inhabitants [103,104,105,106], Taiwan is in position 33 (0.14123%) of the ranking and Catalonia in 702 (0.00929%), and Taiwanese is in position 36 (0.13114%) and Catalan in 135 (0.04125%). Although Catalonia has a higher influx of foreign tourism than Taiwan, there is a marketing problem regarding the promotion of Catalan brands, which confirms findings from previous studies [21]. Table A3 lists the twenty most frequent key terms per region and restaurant category (1, 2, 3 stars). As in Table A2, the keywords that generate the most comments in all categories and regions are food, restaurant, service, and experience. Table 2 also shows the high frequency of comments about wine with respect to the three categories of Catalan restaurants.

### 4.1. Designative Aspect

**Figure 4:**
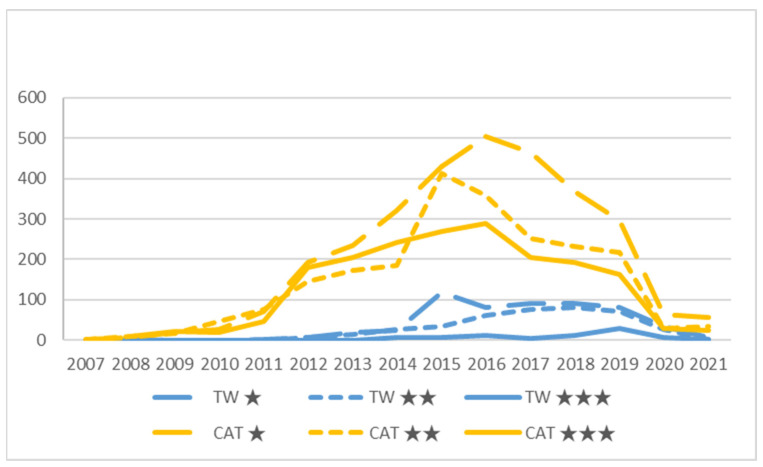
Online travel reviews per categories and year. Source: TripAdvisor OTRs in English (Taiwan: 1038; Catalonia: 7138).

Figure 4 shows the distribution of TripAdvisor OTRs written in English by region and year. Both regions show a sharp drop in OTRs starting in 2020, which is understandable due to the SARS-CoV-2 pandemic lock down. The curves for Taiwan indicate a slight decline from 2018, despite the fact that the influx of foreign tourism from English-speaking countries continued to grow at that time [88]. The curves for Catalonia indicate a decrease in the number of OTRs from 2016; in contrast, English-speaking tourists continued to grow in those years [89], with a slight setback in 2017 due to the serious events that took place (terrorist attack and independence movement) [78,98]. These trends indicate a decline in restaurant popularity as measured by the number of OTRs.

Table 2 shows the descriptive statistics of the temporal distribution of the OTRs by region and restaurant (see Figure 4). Taiwan has 37.18% of restaurants, but only 12.70% of OTRs. Compared with the official statistics of foreign tourists in 2019 and adding the visitors in both regions, Taiwan had 27.96% of the total. Although the kurtosis value is close to three, the skewness value is far from zero in both cases, which shows that the distribution of the data is abnormal. To avoid inconsistencies in the comparisons, the study considers the set of years as a single segment, and the metrics are based on percentages to bridge the differences regarding the number of restaurants and OTRs. Table 3 shows that there is a positive relationship between popularity by number of OTRs and the category of restaurants by Michelin stars.

### 4.2. Appraisive Aspect

Table 3 displays the number of OTRs and the percentage of scores given by customers by region and restaurant category. In all cases, most of the scores are very good or excellent.

Table 4 shows percentages of positive and negative scores, the weighted average of the scores (see Section 3.4.2), and the percentage of terms with positive or negative polarity in relation to total words (including stop words). In all cases, the positive evaluations are much higher than the negative. The aggregation of the five rankings shows a positive relationship between customer satisfaction and restaurant category. In other words, higher-category restaurants provide more well-being to their customers, except in the case of a 3-star restaurant, which is the worst rated. Regarding the regions, in almost all cases the customers value Catalonia’s restaurants better. Considering that English-speaking customers do not have to be more demanding in Taiwan than in Catalonia, nor is there any reason to think that the food in Taiwan restaurants is of inferior quality, there may be marketing problems in relation to the creation of consumer expectations.

### 4.3. Prescriptive Aspect

Table 5 shows the attitudinal and behavioral responses to the previous stimuli by regions and restaurant categories as a percentage of key terms over the total number of words (including stop words). The figures obtained do not allow conclusions to be drawn related to the restaurant categories. However, in almost all cases the results are more positive in Taiwan, which means customers are more loyal in Taiwan than in Catalonia.

### 4.4. Perceived Quality of Restaurant Services

Within the “Technical quality” category in Appendix C, Box A1 shows a sample of consumer comments regarding food. According to the reviews content, consumers who dine in Michelin-starred restaurants mainly focus on the customer experience centered on gastronomy. What they value is the quality of the set meal that includes an appetizer, main dish, side dish, and dessert. Box A2 shows a sample of consumer comments regarding drink. In addition to the main meal, the pairing beverages also play an important role in the customer’s gastronomic experience. High-quality beverages often add value to the food, and they sometimes may also become the protagonist.

Within the category of “Functional quality”, Box A3 includes a sample of consumer comments regarding hardware. Customers who plan to dine in Michelin-starred restaurants have expectations directly proportional to the price; therefore, in terms of hardware provision, the menus, utensils, tables and chairs, and overall decorations must give customers a sense of dignity. In addition, facilities such as lockers and shoe cabinets allow customers to enjoy a more comfortable dining space, which also has a positive impact on customers’ gastronomic experience. Box A4 includes a sample of consumer comments regarding ambience. Even with the same precious hardware facilities and different layouts, different dining atmospheres of space will be created. While paying attention to the quality of the food, customers of Michelin-starred restaurants also consider the dining atmospheres, especially the feeling of the space.

Within the category of “Service performance”, Box A5 shows a sample of consumer comments regarding the chef. In addition to being the soul of the Michelin-starred restaurant, the chef has a certain social status under the blessing of the *Michelin Guide*. Although not every customer cares about interacting with the chef, for many it is important to be able to do so since they come to consider him as a kind of celebrity. Customers are deeply impressed if the chef can personally interact with the guests before, during, and after the meal. Box A6 shows a sample of consumer comments regarding the staff. On the one hand, customers require attentive and high-quality service; but on the other hand, they do not want to be disturbed because the service is too attentive; thus, proper and in-place service is the most suitable.

Within the category of “Perceived service quality”, Box A7 includes a sample of consumer comments regarding image as a quality (product, service, and atmospherics). The recognition of a Michelin-starred restaurant is a “chef-centric” standard in terms of the *Michelin Guide*, but regarding the sustainable management of the restaurant, it must be “Customer-centric”. Therefore, regardless of the food innovation, the creation of the dining environment, the appropriate service, and even the special dietary needs of customers should all be concerned, so that the customers will become repeat customers when leaving with a good gastronomic experience. These are the parts that Michelin-starred restaurant service industries need to pay attention to and achieve.

## 5. Conclusions

The study proposes some conceptual models to determine the subjective well-being of diners through content generated and shared online by the customers of the restaurants considered. The theoretical framework is tested in the case study of Michelin-starred restaurants located in two regions with similar characteristics and different gastronomic cultures –Taiwan (Asia) and Catalonia (Europe). The results show that, overall, customers in both regions were satisfied, with the majority rating the restaurant experience as very good or excellent. The findings confirm the conclusions of a previous study on gastronomic activities in both regions other than restaurant food, in terms of branding and marketing, as well as popularity and valuation of the activities. For example, the preponderance of Catalan wines: since Catalonia’s wine industry has had a long tradition, 6% of total agricultural revenues come from the wine sector [107].

After COVID-19 began (2020), compared to their peak periods (Taiwan, 2015; Catalonia, 2016) the number of tourists dropped to only 40% and 10%, respectively, indicating that the pandemic has had a significant impact on international tourist destinations. In contrast, tourists still like to go to the capital (Taipei and Barcelona) to experience luxurious cuisine. The dishes (especially steaks) and services of the two places have received positive responses from customers.

Although big data technology is not yet mature, it has been widely used in many fields. Compared with traditional research methods, big data has the advantages of a large sample size, simple collection method, and having fewer human interferences. Big data can reduce sampling and system-related errors. However, in the context of practical application, shortcomings remain that cannot completely replace traditional research methods. As far as big data sources are concerned, UGC is a huge and available resource. Based on appropriate conceptual models and methodological frameworks, the data sources can be converted into useful information to achieve the predetermined goal through technology. Users autonomously generate electronic word-of-mouth after their own experiences on social media, and academia takes these data sources to generate valuable research results, through appropriate technologies, to provide industry or government as a reference. Furthermore, the industry or government can improve the original experience or design a new one according to tourists’ preferences or needs to achieve a double-win situation.

A luxury gastronomic experience can increase the tourism flow and become a new indicator of tourist destinations. Catalonia is a popular tourist destination in Europe, and Taiwan is an emerging tourist destination in Asia, each with its own tourist characteristics and attractions. The results show that the number of reviews of Michelin-starred restaurants in the two regions began to decline under frequently appearing Black Swan Events, and continued falling after the appearance of COVID-19. In addition to providing corresponding relief programs, the government should help the industry develop other improvement programs to tide over the difficulties.

### 5.1. Theoretical Implications

The study proposes two new integrated theoretical models to analyze subjective well-being in relation to gastronomy tourism. The theoretical framework is supported by content generated by users or consumers and shared on social media. The first model (Figure 1) is a combination of the hermeneutical circle of image formation [39] and the service quality model [22]. In short, agents project a gastronomic image that generates pre-visit expectations in prospective customers. The on-site experience is impacted by the technical quality, functional quality, and service environment. Then, the customer perceives the image as a quality of the product, service, and atmospherics. This perception of the customer, conditioned by his/her expectations, leads to post-visit feelings of satisfaction and subjective well-being. The study uses an extract (Figure 2) from the model in Figure 1 to qualitatively analyze the factors that influence the gastronomic image perceived by diners in a quality restaurant.

The second model is an adaptation of the semiotic aspects of the tourist destination images [23] to the gastronomic image and well-being of visitors. The model is divided into three distinctly different but hierarchically interrelated semantic aspects—designative, appraisive, and prescriptive—and three pragmatic uses—informative, valuative, and incitive. These triads enable the quantitative analysis of three variables—popularity of the tourist resource and the satisfaction and loyalty of visitors—which are indicators of their well-being in the in situ experience phase.

The importance of the proposed framework lies in the strength of the two integrated conceptual models that enable the qualitative (Figure 1 and Figure 2) and quantitative (Figure 3) methodologies to measure the satisfaction and well-being of tourists resulting from a quality gastronomic experience via UGC as a data source. According to most research, the empirical results have confirmed the positive relationship between perceived service quality and consumer satisfaction and, therefore, their subjective well-being, just as they have revealed a positive relationship between quality and popularity of the service. In addition, the model in Figure 1 is useful for analyzing other quality services in the field of hospitality and tourism, such as luxury hotels.

### 5.2. Managerial Implications

Findings in both regions reveal a positive relationship between the quality of restaurants by the number of Michelin stars and their popularity by number of OTRs. In addition, they confirm the well-known positive relationship between service quality and consumer satisfaction and well-being, except for a three-star restaurant in Taiwan, which was shown to be the least-valued by customers.

Comparison of the results in both regions detected branding deficiencies in Catalonia; the Catalonia and Catalan brands were much less mentioned by English-speaking tourists than the Taiwan and Taiwanese brands. There may also be a marketing flaw when nearly all of the metrics indicating consumer loyalty were more positive in Taiwan. However, Taiwan has to improve marketing strategies because the popularity of restaurants by number of OTRs was lower in proportion to that of Catalonia. Furthermore, almost all the metrics indicating consumer satisfaction were less positive in Taiwan.

The framework applied to Michelin-starred restaurants in Taiwan and Catalonia could also be useful in other tourist destinations at various territorial levels, including cities, countries and regions, as well as in other services of recognized quality, including restaurants classified in the “Global Masters”, “Masters” and “Very Fine” levels in the *White Guide* (whiteguide.com/nordic/en, accessed on 22 February 2022) or the “Luxury”, “First-class”, and “Comfort” hotels according to the standard star rating system.

The proposed methods are relatively easy to implement because they do not require complex statistical tools. Data collection is cost effective, which allows tourism destination management or marketing organizations and other stakeholders to obtain outcomes in near real time from a demand-side perspective and be able to compare them with results in other spots. The information obtained can be useful to design or improve quality products and services.

### 5.3. Limitations and Future Work

Given that the positive relationship between satisfaction and consumer loyalty has been demonstrated by numerous researchers through surveys, the main limitation of the study is that the results on attitudinal and behavioral responses to previous stimuli, namely designative and appraisal, are not conclusive and, therefore, do not quantitatively confirm that satisfaction is a determinant of consumer loyalty. This issue with TripAdvisor OTRs as a data source is consistent with previous research through Airbnb OTRs [23], which was also unable to demonstrate such a relationship. This setback does not directly affect current research because the doctrine agrees that consumer satisfaction is a determinant of subjective well-being. In future work, it will be necessary to refine the categories of the prescriptive aspect of the semiotic model to better collect the key terms that indicate consumer loyalty.

## Figures and Tables

**Table 1:** Ranking aggregation example.

Candidate	X (+)	X Rank	X Points	Y (−)	Y Rank	Y Points	Sum	Rank
C1	18	2	3	25	1	0	3	4
C2	20	1	4	11	5	4	8	1
C3	10	5	0	22	2	1	1	5
C4	12	4	1	13	4	3	4	2.5
C5	15	3	2	16	3	2	4	2.5

**Table 2:** Number of restaurants and English reviews in Taiwan and Catalonia in November 2021.

Region	N	OTRs	Min.	Max.	Mean	Median	Std. Dev	Skew.	Kurt.
TW	29	1038	1	171	35.79	20	42.80	1.70	2.63
CAT	49	7038	5	834	145.67	57	194.59	1.93	3.30

Note. N: number of restaurants; Min.: minimum number of OTRs per restaurant; Max.: maximum; Std. Dev: standard deviation; Skew.: skewness; and Kurt.: kurtosis.

**Table 3:** Number of customer reviews and scores per region and restaurant category.

Region	Category	N	Count	5* (%)	4* (%)	3* (%)	2* (%)	1* (%)
	1 star	21	559	60.11	23.97	8.05	3.40	4.47
TW	2 stars	7	400	62.00	23.00	7.25	3.75	4.00
	3 stars	1	79	59.49	24.05	6.33	5.06	5.06
	1 star	37	3052	69.95	15.27	7.57	4.03	3.18
CAT	2 stars	9	2189	74.60	12.33	6.30	3.52	3.24
	3 stars	3	1897	79.86	9.33	5.38	2.74	2.69

Note. (5–1)* = TripAdvisor score bubble (excellent, very good, average, poor, terrible).

**Table 4:** Average scores and percentage of terms in evaluative and affective dimensions.

Region	Category	Score−	Score+	AvgScore	Feel−	Feel+	Rank
	1 star	7.87	84.08	82.96	0.57	4.38	2
TW	2 stars	7.75	85.00	83.81	0.54	4.21	1
	3 stars	10.13	83.54	81.96	0.59	4.22	3
	1 star	7.21	85.22	86.20	0.59	4.56	3
CAT	2 stars	6.76	86.93	87.88	0.53	4.16	2
	3 stars	5.43	89.19	90.23	0.45	3.73	1

Note. − = negative polarity; + = positive polarity.

**Table 5:** Percentage of key terms in attitudinal (A) and behavioral (B) dimensions.

Region	Category	Behav−	Behav+	Rank B	Recom−	Recom+	Rank A
	1 star	0.0000	0.0381	2	0.0398	0.3528	1
TW	2 stars	0.0041	0.0345	3	0.0406	0.2437	2
	3 stars	0.0000	0.0856	1	0.0428	0.1818	3
	1 star	0.0025	0.0343	2	0.0425	0.3156	1
CAT	2 stars	0.0019	0.0232	2	0.0474	0.2851	2.5
	3 stars	0.0015	0.0198	2	0.0448	0.2115	2.5

Note. − = negative polarity; + = positive polarity.

## Appendix A. Systematic Literature Review

**Table A1:** Thirteen articles indexed in the Web of Science or in Scopus on gastronomy tourism and well-being.

Ref.	Context	Major Observations	Method
[34]	(2022) food neophobic tourists and their well-being	Tourists with food neophobia experienced positive effects on well-being after comfort food consumption. The findings offer insights for guiding future research on food neophobia and comfort food in tourism.	Questionnaire, Harman’s single factor test, ANOVA, Pearson’s correlation analysis
[108]	(2021) wellbeing perceptions among restaurant diners	Public health regulations and social distancing measures impact consumers’ dining experiences and their comfort/discomfort. The domains of wellbeing seem to be very important for the individual, and they influence not only the restaurant choice but also the overall dining experience and the intention to revisit during the COVID-19 era.	Qualitative data obtained from in-depth interviews with consumers, Nvivo
[109]	(2021) employee well-being	The multidimensional nature of employee well-being and the underscored significance of a personal approach to defining employee-wellbeing was confirmed. The most critical dimension of employee well-being identified was workplace experience. Workplace happiness acts as an affective dimension of employee well-being.	interviews and survey, 7-point Likert scale, exploratory factor analysis (EFA), confirmatory factor analysis (CFA)
[110]	(2021) braggart WoM based on co-created aspects of cooking classes	Co-designing the experience contributed to better perceptions of culinary service consumption. Prior knowledge influences consumer perceptions of the experience and its co-creative aspects.	Sequential mixed-method approach, semi-structured interviews, post-experience survey, interactive co-design approaches, qualitative and quantitative approach
[111]	(2021) halal food anxiety	Halal food anxiety was positively associated with pandemic travel anxiety but negatively related to the psychological well-being of Muslim modelling. Various avenues are highlighted to exploit the vast commercial halal food market in non-Muslim majority destinations.	Questionnaire survey, structural equation modelling (SEM), confirmatory factor analysis (CFA)
[112]	(2021) neophobia and well-being	The results show that having an authentic food experience is most strongly associated with the sense of meaningfulness of the trip and experiencing positive emotions during the trip. Enduring food involvement can explain both hedonic and eudaimonic aspects of the degree of the impact of food experiences on well-being.	Questionnaires, covariate-based structural equation modelling (CB-SEM), squares structural equation modelling (PLS-SEM), measurement model common method variance (CMV), Harman’s one-factor test
[33]	(2020) well-being of foodies	Taiwan food possesses different values for mainland Chinese. A significant positive correlation was observed between food consumption motivations and food experiential values. Value for money, service excellence, aesthetics, and escapism are likely to influence foodies’ well-being.	Questionnaire survey, structural equation modelling (SEM), confirmatory factor analysis (CFA)
[113]	(2020) older adults and sources of wellbeing	Through social interaction and leisure activities, casual restaurant environments (unassuming third places) become possible wellbeing places, including local coffee shops and fast food restaurants; health and well-being can be supported through stimulation, support, protection, and care mechanisms.	Thematic analysis of interviews, qualitative interviews, and ethnographic fieldwork with quantitative survey data, multilevel linear regression model, Nvivo
[114]	(2020) customer mistreatment and employee well-being	Customers will vent to the restaurant staff of their unsatisfactory daily life, which will affect their well-being. Poor well-being is caused by stressors in the workplace through a lack of psychological detachment from work at home. Employees are more effectively able to cope with work stressors when they have sufficient personal resources.	Survey, multilevel confirmatory factor analysis (CFA), eight-factor model, two-level hierarchical linear modelling (HLM)
[115]	(2020) food safety knowledge and hygienic-sanitary control	Keen attention must be paid to ensuring adequate and proper flow of knowledge among handlers to ensure that proper hygiene-related standards are adhered to with utmost rigidity, and ensure they are well-motivated to practice hygienic sanitary controls.	Questionnaire, 5-point Likert scale, confirmatory factor analysis (CFA), structural equation model (SEM), Herman’s single factor test
[32]	(2017) travelers’ holiday well-being	A destination’s gastronomy effect on well-being is founded on local eating habits, traditions, safety, and locally produced food and drinks (wine, beer, and juice) in line with the destinations’ culture. Even though some travelers only consume food for survival, most food and eating activities contribute to holiday well-being.	Quantitative research approach, questionnaires, 4-point Likert-type scale, univariate analysis (t-test, ANOVA, and regression analysis)
[116]	(2015) community well-being	Establish a teaching and learning center for indigenous and cultural tourism in a tertiary education institution to educate locals and tourists. The specific roles of chefs and other hospitality personnel were highlighted, including their relevance in the hospitality and tourism industries.	Scrutiny of available literature
[11]	(2014) psychological well-being	Cuisine experience and psychological well-being influence hot springs tourists’ revisit intentions and only cuisine experience affects psychological well-being; however, the significance of these factors varied based on the self-health perception levels (high or low) of tourists in the sample.	Questionnaire, 5-point Likert-type scale, confirmatory factor analysis (CFA)

## Appendix B. Key Terms Frequency

**Table A2:** Forty most frequent key terms per region.

	Taiwan		Catalonia			Taiwan		Catalonia	
Rank	Key Term	%	Key Term	%	Rank	Key Term	%	Key Term	%
**1**	food	0.68680	food	0.68774	**21**	nice	0.17906	dish	0.16861
**2**	restaurant	0.57668	restaurant	0.59574	**22**	dinner	0.17738	really	0.16072
**3**	good	0.51952	menu	0.51503	**23**	dish	0.17653	time	0.16022
**4**	service	0.50018	wine/s	0.51014	**24**	chef	0.17233	Michelin	0.15103
**5**	great	0.40519	service	0.45090	**25**	delicious	0.15468	dinner	0.14584
**6**	experience	0.32197	experience	0.45070	**26**	steak	0.15468	courses	0.13835
**7**	Taipei	0.29675	good	0.31275	**27**	table	0.15047	restaurants	0.13585
**8**	menu	0.26732	great	0.29927	**28**	quality	0.14795	table	0.13575
**9**	dishes	0.26564	best	0.24553	**29**	really	0.14795	dining	0.13345
**10**	best	0.25640	meal	0.24423	**30**	price	0.14375	chef	0.12376
**11**	place	0.24715	dishes	0.24093	**31**	course	0.14291	nice	0.11957
**12**	staff	0.22949	tasting	0.22665	**32**	amazing	0.14123	delicious	0.11807
**13**	wine/s	0.22529	amazing	0.20967	**33**	Taiwan	0.14123	worth	0.11497
**14**	excellent	0.22025	course	0.20797	**34**	lunch	0.13534	lunch	0.11317
**15**	time	0.21689	Barcelona	0.19838	**35**	reservation	0.13198	taste	0.10938
**16**	just	0.19419	excellent	0.19708	**36**	Taiwanese	0.13114	star	0.10798
**17**	meal	0.18998	staff	0.19648	**37**	try	0.12862	perfect	0.09999
**18**	dining	0.18662	just	0.19388	**38**	set	0.1261	went	0.09809
**19**	beef	0.18326	like	0.17650	**39**	taste	0.12105	kitchen	0.09789
**20**	like	0.18326	place	0.17291	**40**	served	0.11853	wonderful	0.09779

Note. Total words (including stop words): TW = 118,957; CAT = 1,001,109. Unique words: TW = 6265; CAT = 18,895.

**Table A3:** Twenty most frequent key terms per region and restaurant category.

	Taiwan		Taiwan		Taiwan		Catalonia		Catalonia		Catalonia	
Rank	1-Star	%	2-Star	%	3-Star	%	1-Star	%	2-Star	%	3-Star	%
1	food	0.65	food	0.75	service	0.66	food	0.78	food	0.69	food	0.58
2	good	0.62	restaurant	0.57	food	0.63	restaurant	0.67	restaurant	0.57	restaurant	0.54
3	restaurant	0.58	service	0.50	restaurant	0.58	menu	0.57	menu	0.55	wine/s	0.54
4	service	0.47	great	0.43	hotel	0.56	service	0.50	wine/s	0.55	experience	0.50
5	great	0.40	good	0.42	duck	0.51	wine/s	0.45	experience	0.50	menu	0.42
6	place	0.33	experience	0.41	good	0.44	good	0.42	service	0.45	service	0.40
7	Taipei	0.33	wine/s	0.35	experience	0.43	great	0.37	great	0.30	best	0.29
8	best	0.31	dishes	0.32	dishes	0.31	experience	0.36	meal	0.29	meal	0.25
9	beef	0.31	menu	0.32	great	0.31	excellent	0.27	tasting	0.29	great	0.22
10	steak	0.29	Taipei	0.26	menu	0.31	dishes	0.26	good	0.28	good	0.22
11	staff	0.26	dining	0.25	excellent	0.30	best	0.24	course	0.26	just	0.22
12	experience	0.23	dish	0.24	pork	0.28	place	0.23	dishes	0.26	course	0.22
13	excellent	0.23	time	0.24	Taipei	0.28	tasting	0.22	Barcelona	0.24	dishes	0.21
14	menu	0.22	chef	0.23	palais	0.26	Barcelona	0.21	staff	0.22	amazing	0.21
15	dishes	0.21	just	0.22	best	0.25	amazing	0.20	amazing	0.22	like	0.19
16	quality	0.20	course	0.20	nice	0.22	meal	0.20	best	0.21	world	0.18
17	time	0.20	staff	0.20	dining	0.21	Michelin	0.19	just	0.19	staff	0.18
18	meat	0.20	excellent	0.20	just	0.21	staff	0.19	dish	0.18	tasting	0.18
19	nice	0.19	like	0.20	room	0.21	just	0.18	excellent	0.18	table	0.17
20	meal	0.19	lunch	0.20	time	0.21	dish	0.17	like	0.18	time	0.16

Note. % = Percentage of the total words in the category (including stop words).

## Appendix C. Sample of Consumer Comments

Box A1 Sample of consumer comments regarding food. Positive: "*One thing that particularly impressed me was all the variety in how the dishes are presented. They put a lot of effort in making how the food comes to you part of it, and in bringing it in a lot of different kind of plates. In terms of actual dishes, they are all small tasting portions, and they better be, as there are around 17 of them to try. We got a fatty tuna on top of a tuna bone that just melted in your mouth. The prawn head with a sort of prawn infused rice was also hard to forget. I enjoyed the fish dishes a bit more than the meat ones, but the duck was also awesome. The desserts were extremely fresh and interesting to close it out, although at that point it was hard to eat anything else after all that awesomeness*". Negative: "*Overall experience was poor, from the menu content and taste, to food presentation and environment We have been to many 1, 2 and 3 Michelin starred restaurants around Europe. This one does not deserve it’s stars. We tried the white truffle menu. What a disappointment! Beyond the fact that white truffle was bearably visible on each dish (although we ordered the more expensive truffle menu), most dishes look uninviting and tasted the same. While mushrooms with truffle pairs well, a couple of the dishes had that combination and with the additional fact that little real truffle was served, most tasted mainly of mushrooms! Dessert was equally disappointing, a very strange combination of tastes. We left our plates quite untouched*".

Box A2 Sample of consumer comments regarding drink. Positive: "*We decided to have the wine pairing and my daughter had the tea pairing. Each dish we had was outstanding, absolutely superb. The wine pairing was excellent and the tea pairing very information*". Negative: "*My husband got a wine pairing with his food. It was the cheapest wine you could get...and pairing? They can’t even choose the correct wine to the type of food they are serving. He had to return a few glasses, there was either a cork in the glass (like lots of small pieces) or it didn’t even match with the food! We were really disappointed*".

Box A3 Sample of consumer comments regarding hardware. Positive: "*From small touches such as a beautiful wooden locker with your own key to keep handbags and coats so they don’t smell from the grilled meats*". Negative: "*Plain environment and decor plus uncomfortable chairs added to our disappointment*".

Box A4 Sample of consumer comments regarding ambience. Positive: "*The design is modern, clean lines, very pleasant and the ambience was upbeat and a very nice vibe complemented with the just right lighting*". Negative: "*Good and tasty food. But the place is tiny, no decoration whatsoever, not even a painting on the wall. The ambiance was lousy as the restaurant was full and there was no music or windows so the noise was terrible. The waiters were not even polite as they were stressed running from one table to another. Poor experience for the price*".

Box A5 Sample of consumer comments regarding chef. Positive: "*The beef, chicken, pork grill is exceptionally good, and the chef is really funny that we had a very nice talk. It may not be a good idea to grill scallop and prawn (as the chef suggested), I would recommend to eat them raw if they are fresh to retain the original taste, grilling is not a good cooking method, I would suggest to order red meat in here*". Negative: "*The Chef came to greet only 2 tables (probably clients that he knew). I think it’s a pity because for a Michelin star restaurant, it’s the first time the Chef did not come to greet his clients, especially when the restaurant is small. I would have been pleased to exchange with him (I do speak Spanish fluently so the language was not a barrier)*".

Box A6 Sample of consumer comments regarding staff. Positive: "*Food was superb and great quality. Service perfect—we booked by phone a few hours prior. Staff members spoke English and engaged well with us (they also knew when to stop engaging to give privacy). We discovered that they all take Japanese lessons, and make the effort to learn common languages of customers. Dishes cooked meticulously with specific technique for each, and the mesh is changed in between every dish. Price reasonable. Very impressed*". Negative: "*I understand that service staff must have been busy and tired. Can’t help but noticed the lack of enthusiasm from manager and staff right from the point of receiving menu. While taking orders, the monotonous, matter of fact, quickly order and get it over and done with attitude was a wet blanket. There was no smile. Neither was there an attempt to explain the different beef offerings and other main courses*".

Box A7 Sample of consumer comments regarding perceived service quality (product, service, and atmospherics). Positive: "*I think it’s still worth to pay a visit. I love the interior design, wood is the main frame with modern look. Food is undoubtly perfect on the plate and the taste. Staff there are very presentable that they would explain what you’re eating. Recommend the cocktail. The one I ordered is very special and unforgettable. Although I forget the name, the curry powder on the glass was attractive. 2 options for the menu, smaller or full course. I must say I regret to pick the full course. It really made me too full and I had some left on the plate for the last 3 dishes*". Negative: "*The food is presented beautifully. However, they forgot the most basic thing: it needs to be tasty! And it is just not. We took the 12 courses tasting menu with wine tasting. We were served with cold bread that although wasn’t stale, was getting there. Unacceptable in a supposedly 2 Michelin stars restaurant. Some of the dishes were just ok but hardly interesting or special. Meh is the word I’d use for those. Others failed badly on flavours: a too salty demi glasse sauce or a combination that just don’t work like cheese and creme dessert with white truffles which was awful. The wine pairing also didn’t match most dishes. Who drinks Saki with a dish that has Thai like coconut curry? It appears that most if the waiters there have only basic English and partial knowledge of what they are serving. For a lot of our questions they had to call the head waiter to explain or answer. Service is extremely slow, even for a fancy gourmet restaurant. On some courses we got the paired wine but then waited over 30 min for the dish itself. At the end we payed almost 400 euros for a bad dinner. We felt deceived and robbed. This place can’t even compete with small tapas bars that serve wonderful tasting food*".
